# Protective Effect of Ginsenoside Rg1 on Oxidative Damage Induced by Hydrogen Peroxide in Chicken Splenic Lymphocytes

**DOI:** 10.1155/2019/8465030

**Published:** 2019-04-18

**Authors:** Shicheng Bi, Xiaodan Ma, Yuemin Wang, Xiaoqing Chi, Yong Zhang, Wei Xu, Songhua Hu

**Affiliations:** Department of Veterinary Medicine, College of Animal Sciences, Zhejiang University, Hangzhou, Zhejiang 310058, China

## Abstract

Previous investigation showed that ginsenoside Rg1 (Rg1) extracted from *Panax ginseng* C.A. Mey has antioxidative effect on oxidative stress in chickens. The present study was designed to investigate the protective effects of Rg1 on chicken lymphocytes against hydrogen peroxide-induced oxidative stress and the potential mechanisms. Cell viability, apoptotic cells, malondialdehyde, activity of superoxide dismutase, mitochondrial membrane potential, and [Ca^2+^]i concentration were measured, and transcriptome analysis and quantitative real-time polymerase chain reaction were used to investigate the effect of Rg1 on gene expression of the cells. The results showed that treatment of lymphocytes with H_2_O_2_ induced oxidative stress and apoptosis. However, pretreatment of the cells with Rg1 dramatically enhanced cell viability, reduced apoptotic cells, and decreased oxidative stress induced by H_2_O_2_. In addition, Rg1 reduced these H_2_O_2_-dependent decreases in mitochondrial membrane potential and reversed [Ca^2+^]i overload. Transcriptome analysis showed that 323 genes were downregulated and 105 genes were upregulated in Rg1-treated cells. The differentially expressed genes were involved in Toll-like receptors, peroxisome proliferator-activated receptor signaling pathway, and cytokine-cytokine receptor interaction. The present study indicated that Rg1 may act as an antioxidative agent to protect cell damage caused by oxidative stress via regulating expression of genes such as RELT, EDA2R, and TLR4.

## 1. Introduction

In the modern poultry industry, animals usually suffer from highly condensed population, contaminated feed, pollutant air, and inappropriate management, which may result in oxidative stress and lead to immunity dysfunction [1]. For example, consumption of aflatoxin B1 diets was reported to significantly inhibit the immune responses of chickens to vaccination against Newcastle disease [2]. Another study suggested that aflatoxin B1 increases oxidative stress, induces excessive apoptosis of lymphocytes in the spleen and bursa, and decreases the immunity [3, 4]. Environmental pollutants such as cadmium and H_2_S were reported to be toxic on the spleen or other organs of chickens, have negative effect on the immunity, cause oxidative stress, and consequently exacerbate disharmony of the immune and antioxidative systems [5, 6]. Though the exact mechanism is complicated, unbalance between reactive oxygen species (ROS) production and elimination has been widely implicated for the damage of the immune system and immunodeficiency [7, 8]. As a peripheral immune organ, the spleen is one of the principal sites for priming of the primary immune responses [9]. Accumulating literatures have reported that oxidative stress is associated with suppressed immune function in animals [10, 11].

Previous investigation showed that saponins extracted from the stem and leaf of *Panax ginseng* C.A. Mey (GSLS) have antistress effect on chickens [12]. More than 30 ginsenosides have been identified from the herb as of yet, and ginsenoside Rg1 (Rg1), a steroidal saponin, is one of the active constituents in GSLS [13, 14]. The saponin was found to display antioxidant activity in mice and human [15, 16]. It was reported that Rg1 had an antioxidant effect by alleviating oxidative damage in a cardiomyocyte hypoxia/reoxygenation (H/R) model [17]. Liu et al. showed that Rg1 can prevent apoptosis and ROS production in oxidative modification of human umbilical cord blood-derived stromal cells induced by *tert*-butyl hydroperoxide [18]. Very recently, oral administration of Rg1 was reported to have effects of antioxidative stress and immunomodulation in chickens [19].

In 2017, the Agricultural Ministry of China issued a certificate approving the product made from GSLS to be used in poultry. However, very few reports have been found regarding the mechanisms underlying the antistress in chickens. The present study was designed to investigate the protective effects of Rg1 on chicken splenic lymphocytes treated with hydrogen peroxide (H_2_O_2_). H_2_O_2_ is often experimentally used to stimulate production of ROS in vitro [20, 21]. RNA sequencing (RNA-seq) is a technique to quantify differentially expressed genes involved in various biological processes. The possible molecular mechanisms were explored at the transcriptome level by RNA-seq analysis, and the DEGs of interest were validated by quantitative real-time polymerase chain reaction (RT-qPCR).

## 2. Materials and Methods

### 2.1. Preparation of Chicken Splenic Lymphocytes

The procedures on handling animals in this experiment were approved by the Institutional Animal Care and Use Committee of Zhejiang University. The process was mainly performed as previously described [22]. Briefly, the spleens were dissected from 30 d old Sanhuang broilers (female) (Ningbo Zhenning Stock Breeding Inc., Ningbo, China) and polished to homogenate. Single cell suspension was obtained by gently pushing the homogenate through a 70 *μ*m sterile plastic mesh. Cells were washed twice and then layered over equivalent lymphocyte separation medium (Tian Jin Hao Yang Biological Manufacture Co. Ltd., Tianjin, China). The suspension was centrifuged at 2,000 × g for 15 min at room temperature, and a white interface was obtained. Then, the cells were washed twice and cultured in RPMI 1640 (Genom Biotech Co., Hangzhou, China) containing HEPES and 2 mM glutamine, supplemented with 10% FBS (Sijiqing Co., Hangzhou, China). Splenic lymphocyte density was adjusted to 5 × 10^6^ cells/mL, and the survival rate of the freshly obtained cells was more than 95% (trypan blue exclusion test) [23].

### 2.2. Cell Treatment

Cells were seeded in 6-well plates (5 × 10^6^ per well), added with Rg1 solution (Puruifa, Chengdu, China) at final concentrations of 0, 50, 70, and 90 *μ*g/mL, respectively, and then cultured for 24 h. The concentrations of ginsenoside Rg1 were used based on the previous study [24]. After that, cells were washed twice with PBS and incubated in media containing H_2_O_2_ (100 *μ*mol/L) for an additional 4 h. Cells treated with H_2_O_2_ only were used as a model, and cells without any treatment were used as a control (Table 1). Finally, the cells were collected for analyses of redox parameters, cell viability, cell apoptosis, mitochondria transmembrane potential, and [Ca^2+^]i concentration.

### 2.3. Biochemical Determinations

The method was used as previously described [21]. To release the intracellular content, 1 mL of cell suspension (5 × 10^6^ cells) was sonicated and centrifuged at 1,000 × g for 5 min. The content of the cell proteins was measured with a bicinchoninic acid kit (BCA) (Beyotime Co., Jiangsu, China). Content of malondialdehyde (MDA) and activity of superoxide dismutase (SOD) were measured with spectrophotometric assay kits (Nanjing Jiancheng Institute of Bioengineering and Technology, Nanjing, China). All samples were analyzed in triplicate.

### 2.4. Determination of Intracellular ROS Production

ROS generation was measured by a reactive oxygen species assay kit (Yeasen Biotech Co. Ltd., Shanghai, China) with 2,7-dichlorodihydrofluorescein diacetate (DCFH-DA) as a fluorescent probe [25]. Cells (1 × 10^5^) were suspended in 500 *μ*L of DCFH-DA (1 : 2,000 dilutions) with incubation at 37°C for 30 min. The culture was centrifuged at 1,000 × g for 5 min, and the supernatant was discarded. Then, cells were washed twice with PBS and used for flow cytometric (FCM, BD FACSCalibur) analysis. The mean fluorescent intensity (MFI) of intracellular 2,7-dichlorodihydrofluorescein (DCF) was detected by FCM, and the data were analyzed using FlowJo V10 software.

### 2.5. Cell Viability

Live cells were measured by the 3-(4,5-dimethyl-2-thiazolyl)-2,5-diphenyl-2-H-tetrazolium bromide (MTT) assay (Solarbio Co., Beijing, China) using absorbance of formazan in cell lysates according to previous description with modification [26]. Briefly, cells in 96-well plates (5 × 10^4^ cells/well) were incubated for 24 h with Rg1 at final concentrations of 0, 50, 70, and 90 *μ*g/mL, respectively, and then centrifuged at 2,000 × g for 10 min. Then, cells were washed twice with PBS and exposed to fresh media containing H_2_O_2_ (100 *μ*mol/L) for an additional 4 h. Cells treated with H_2_O_2_ only were used as the model, and cells without any treatment were used as the control. After that, cells in each well were incubated with 5 mg/mL MTT for 4 h. Then, the plates were centrifuged at 1,000 × g for 10 min, and the MTT formazan was solubilized in 150 *μ*L dimethyl sulfoxide. The optical density (OD) was read at 570 nm. All tests were carried out in triplicate.

### 2.6. Quantification of Cell Apoptosis

Apoptosis was investigated using an Annexin V-FITC Apoptosis Detection kit (BD Biosciences, San Jose, CA, USA). Cells (1 × 10^5^) were suspended in 195 *μ*L of specific binding buffer with 5 *μ*L Annexin V-FITC and incubated for 10 min at 25°C. Then, the cells were washed twice with phosphate-buffered saline (PBS) and resuspended in 190 *μ*L binding buffer with 10 *μ*L propidium iodide (PI) [27]. Fluorescence in cells was detected by FCM, and the data were analyzed using FlowJo V10 software. Annexin V+/PI- cells were considered as early apoptotic cells.

### 2.7. Measurement of Mitochondrial Membrane Potential

5,5′,6,6′-Tetrachloro-1,1′,3,3′-tetraethyl-imidacarbocyanine iodide (JC-1) is able to enter mitochondria and is widely used as a mitochondrial membrane potential-sensitive dye [28]. The detection was performed according to a manufacturer's protocol (Solarbio Co., Beijing, China). In brief, cells (1 × 10^5^) were incubated with JC-1 (5 *μ*g/mL) for 20 min at 37°C. After washing twice in PBS, JC-1 polymer MFI and monomer MFI were assayed by FCM within 30 min and analyzed using FlowJo V10 software. Mitochondrial depolarization was presented by a reduction in the polymer MFI/monomer MFI.

### 2.8. Observation and Analysis of [Ca^2+^]i

[Ca^2+^]i in lymphocytes were determined as previously reported [22, 29]. Briefly, cells (1 × 10^5^) were incubated with Fluo-3/AM (5 *μ*M) (Solarbio Co., Beijing, China) at 37°C for 30 min. After washing with PBS, cells were observed, and the images were acquired using a fluorescence microscopy equipped with an FITC filter (Nikon Ti-FL; Nikon Cooperation, Japan). For quantitative analysis, the fluorescent signals reflecting the [Ca^2+^]i level were measured by FCM, and the data were analyzed using FlowJo V10 software. Intracellular [Ca^2+^]i was reflected by Fluo-3 fluorescent intensity.

### 2.9. Transcriptome Analysis

Each of the three samples from the model and Rg1 groups was used for the RNA-seq. RNAiso™ Plus (Takara, Dalian, China) was used to isolate the total RNA from cells, according to the manufacturer's instructions. A NanoPhotometer® spectrophotometer (Implen, CA, USA) was employed to determine the RNA purity [30]. A RNA 6000 Nano Assay Kit was employed to check RNA integrity on the Bioanalyzer 2100 system (Agilent Technologies, CA, USA). Transcriptome sequencing, sequence assembly, and data analysis are provided by Novogene Bioinformatics Technology Co. Ltd. (Beijing, China). A total of 2 *μ*g RNA from each sample was employed to construct libraries with a NEBNext® Ultra™ RNA Library Prep Kit for Illumina® (NEB, USA). In brief, poly-T oligoattached magnetic beads were used to extract mRNA from total RNA [31]. NEBNext First Strand Synthesis Reaction Buffer (5x) was used to perform fragmentation. Random hexamer primer and M-MuLV Reverse Transcriptase (RNase H^−^) were used to synthesize first-strand cDNA. Next, DNA polymerase I and RNase H were used to synthesize second-strand cDNA [32]. After that, TruSeq PE Cluster Kit v3-cBot-HS (Illumina) was employed to carry out the cluster of the index-coded samples [33]. Then, the sequencing was executed on an Illumina platform, and 150 bp paired-end reads were produced. Reference genome and gene model annotation files were downloaded from the genome website (ftp://ftp.ncbi.nlm.nih.gov/genomes/all/GCF/000/002/315/GCF_000002315.4_Gallus_gallus-5.0/GCF_000002315.4_Gallus_gallus-5.0_genomic.fna.gz). HISAT2 (v2.0.5) was used to build the index of the reference genome, and paired-end clean reads were aligned to the reference genome (ftp://ftp.ncbi.nlm.nih.gov/genomes/all/GCF/000/002/315/GCF_000002315.4_Gallus_gallus-5.0/GCF_000002315.4_Gallus_gallus-5.0_genomic.gff.gz). HTSeq v0.6.0 was used to count the reads mapped to each gene in samples, and reads per kilobase transcriptome per million mapped reads (RPKM) of each gene were then calculated to estimate the expression level of genes in each sample [34]. The DESeq R (1.16.1) package was selected to investigate the differential expression between the model group and the Rg1 group. A model of DESeq based on the negative binomial distribution was used to determine differential expression genes (DEGs) [35]. The *P* value was assigned to each gene. Genes with *P* < 0.05 and fold change ≥ 1.3 were defined as DEGs [36–38]. A clusterProfiler R package was used to perform Gene Ontology (GO) enrichment analysis and to test the statistical enrichment of differential expression genes in the Kyoto encyclopedia of genes and genomes (KEGG) pathways. GO terms and KEGG terms with *P* value less than 0.05 were considered significantly enriched by DEGs [39, 40].

### 2.10. Real-Time Quantitative PCR Validation

Five DEGs that were upregulated and twelve DEGs that were downregulated in the comparison of Rg1 vs. the model were selected to validate the transcriptome sequencing results using RT-qPCR. PrimeScript™ RT Master Mix (Takara, Dalian, China) was used to convert RNA into cDNA on a T100™ thermal cycler (Bio-Rad, USA) [35]. Sequences of primers used for RT-qPCR were designed using the NCBI primer designing tool (http://www.ncbi.nlm.nih.gov/tools/primer-blast/) and provided in Supplementary Materials: Table S3. The chicken *β*-actin was served as the internal control gene. RT-qPCR with SYBR® Premix Ex Taq™ II (Tli RNase H Plus) (Takara, Dalian, China) on selected genes was carried out on a Multiple Real-Time PCR System (Bio-Rad, USA). A relative quantitative method (2^−ΔΔCT^) was employed to evaluate the quantitative variation [41]. All samples were analyzed in triplicate.

### 2.11. Statistical Analysis

The data were analyzed with one-way ANOVA of SPSS 22.0 (IBM), and the results were expressed as mean ± standard error (S.E.). Duncan's test was used to evaluate the differences among various groups. *P* < 0.05 or <0.01 was considered statistically significant. R software (1.16.1) was used to assess results from RNA-seq.

## 3. Results

### 3.1. Effect of Rg1 on the Redox State and Cell Viability

**Figure 1:**
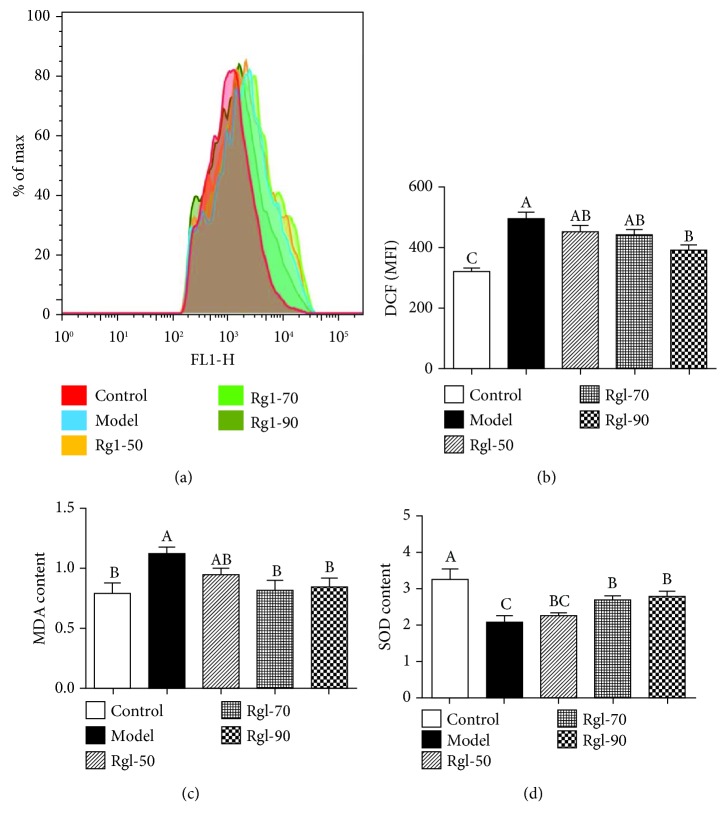
Effect of Rg1 on the redox state within cells. Cells (5 × 10^6^) were treated with Rg1 (0, 50, 70, and 90 *μ*g/mL) for 24 h first and then incubated in media with (model) or without (control) H_2_O_2_ (100 *μ*mol/L) for an additional 4 h. The original tracings showing the (a) DCF fluorescence intensity (intracellular ROS), (b) mean fluorescence intensity (MFI), (c) MDA content, and (d) SOD activities were determined as described in the text. All data are presented as mean ± S.E. (*n* = 6). Bars with different letters were significantly different (*P* < 0.05).

**Figure 2:**
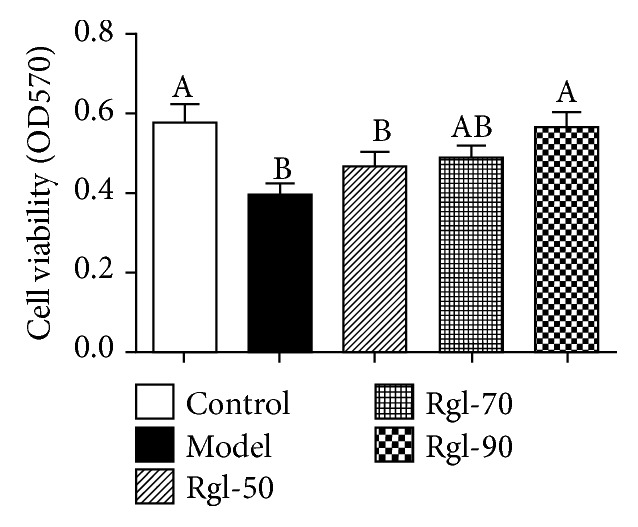
Effect of Rg1 on cell viability. Cells (5 × 10^5^) were treated with Rg1 (0, 50, 70, and 90 *μ*g/mL) for 24 h first and then incubated in media with (model) or without (control) H_2_O_2_ (100 *μ*mol/L) for an additional 4 h. Live cells were measured by the MTT assay using absorbance of formazan in cell lysates. All data are presented as mean ± S.E. (*n* = 6). Bars with different letters were significantly different (*P* < 0.05).

Figure 1(a) showed that cells treated differently had different intracellular DCF fluorescence intensities.  Figure 1(b) showed that cells treated with H_2_O_2_ (model) had a significantly higher intracellular ROS level than the cells without treatment (control) (*P* < 0.01). However, treatment with Rg1 (90 *μ*g/mL) significantly reduced intracellular ROS production when compared with the model (*P* < 0.05). Figures 1(c) and 1(d) showed that cells treated with H_2_O_2_ (model) had significantly higher MDA (*P* < 0.05) and lower T-SOD production (*P* < 0.05) than the cells without treatment (control). Meanwhile, treatment with Rg1 (70 and 90 *μ*g/mL) significantly decreased MDA and increased T-SOD production (*P* < 0.05) when compared to the model.  Figure 2 showed that cells treated with H_2_O_2_ (model) had a significantly lower cell viability than the control (*P* < 0.01), and treatment with Rg1 (90 *μ*g/mL) significantly enhanced cell viability when compared to the model (*P* < 0.05).

### 3.2. Cell Apoptosis

**Figure 3:**
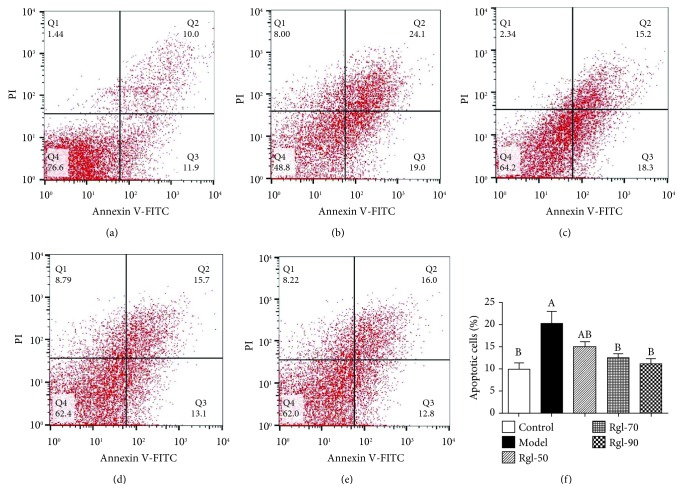
Effect of Rg1 on apoptosis of cells. Cells (5 × 10^6^) were treated with Rg1 (0, 50, 70, and 90 *μ*g/mL) for 24 h first and then incubated in media with (model) or without (control) H_2_O_2_ (100 *μ*mol/L) for an additional 4 h. Apoptotic cells (Annexin V+/PI-) were discriminated by FCM analysis: (a) control group; (b) model group; (c) 50 *μ*g/mL Rg1 group; (d) 70 *μ*g/mL Rg1 group; (e) 90 *μ*g/mL Rg1 group; (f) bar diagram representing apoptotic cell populations. All data are presented as mean ± S.E. (*n* = 6). Bars with different letters were significantly different (*P* < 0.05).

Figures 3(a)–3(e) showed that cells treated differently had different percentages of early apoptotic cells (Annexin V positive and PI negative).  Figure 3(f) showed that cells treated with H_2_O_2_ (model) had significantly increased percentage of early apoptotic cells than the control (*P* < 0.01), and treatment with Rg1 (70 and 90 *μ*g/mL) significantly decreased percentage of early apoptotic cells when compared to the model (*P* < 0.05).

### 3.3. Mitochondrial Membrane Potential

**Figure 4:**
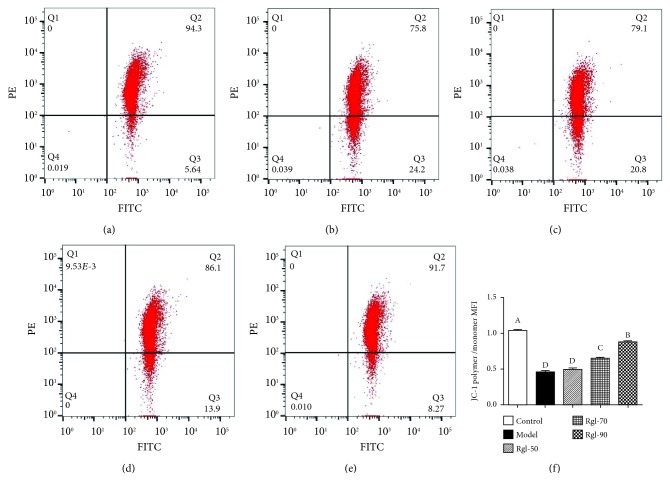
Effect of Rg1 on mitochondrial depolarization. Cells (5 × 10^6^) were treated with Rg1 (0, 50, 70, and 90 *μ*g/mL) for 24 h first and then incubated in media with (model) or without (control) H_2_O_2_ (100 *μ*mol/L) for an additional 4 h. After that, cells (1 × 10^5^) were incubated with JC-1 (5 *μ*g/mL) and assayed by FCM. Mitochondrial depolarization was presented by a reduction in the red/green fluorescence intensity ratio: (a) control group; (b) model group; (c) 50 *μ*g/mL Rg1 group; (d) 70 *μ*g/mL Rg1 group; (e) 90 *μ*g/mL Rg1 group; (f) bar diagram representing JC-1 polymer/monomer MFI. All data are presented as mean ± S.E. (*n* = 6). Bars with different letters were significantly different (*P* < 0.01).

Figures 4(a)–4(e) displayed that cells treated differently had different JC-1 polymer/monomer MFI.  Figure 4(f) showed that cells treated with H_2_O_2_ (model) had significantly decreased JC-1 polymer/monomer MFI than the control (*P* < 0.01). Interestingly, treatment with Rg1 (70 and 90 *μ*g/mL) significantly increased this parameter when compared to the model (*P* < 0.01).

### 3.4. [Ca^2+^]i

**Figure 5:**
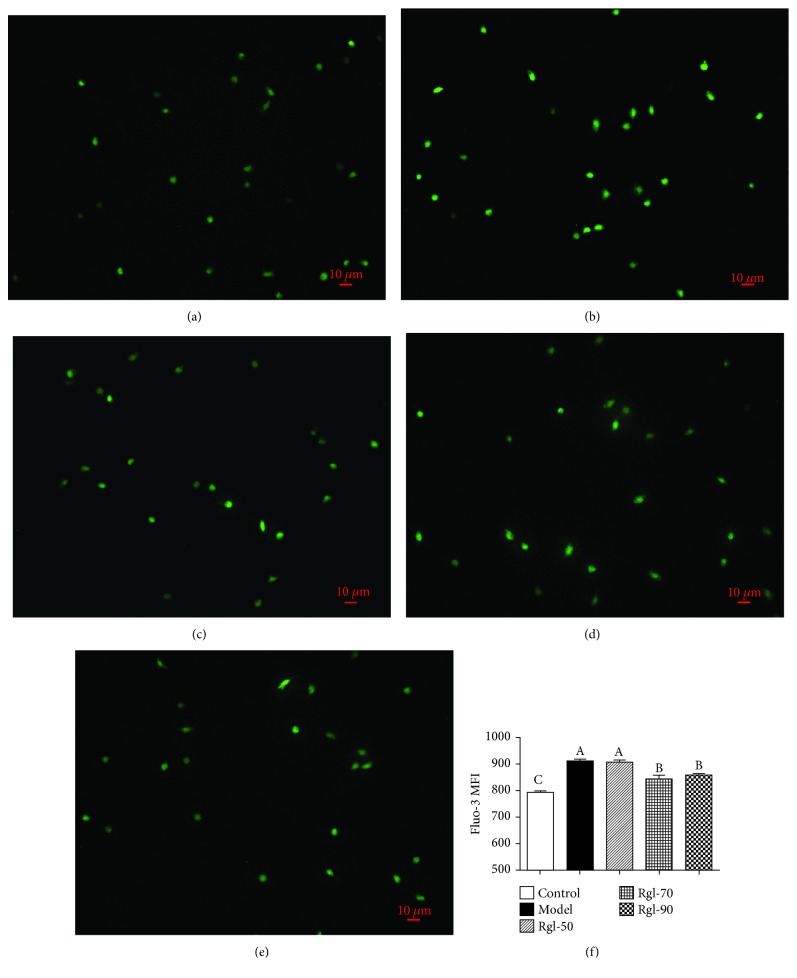
Effects of Rg1 on H_2_O_2_-induced changes in the [Ca^2+^]i levels. Cells (5 × 10^6^) were treated with Rg1 (0, 50, 70, and 90 *μ*g/mL) for 24 h first and then incubated in media with (model) or without (control) H_2_O_2_ (100 *μ*mol/L) for an additional 4 h. After incubation with Fluo-3/AM ([Ca^2+^]i probe), cells were directly observed under a microscope and Fluo-3/AM fluorescence was measured by FCM: (a) control group; (b) model group; (c) 50 *μ*g/mL Rg1 group; (d) 70 *μ*g/mL Rg1 group; (e) 90 *μ*g/mL Rg1 group; (f) bar diagram representing Fluo-3 MFI. All data are presented as mean ± S.E. (*n* = 6). Bars with different letters were significantly different (*P* < 0.05).

The green fluorescence intensity represented the [Ca^2+^]i concentrations in lymphocytes. Figures 5(a)–5(e) showed that cells treated differently had different fluorescence intensities. We also detected fluorescence intensity by FCM. The results implied a similar trend.  Figure 5(f) showed that cells treated with H_2_O_2_ (model) had significantly enhanced green fluorescence intensity than the control (*P* < 0.01). Interestingly, treatment with Rg1 (70 and 90 *μ*g/mL) significantly reduced green fluorescence intensity when compared to the model (*P* < 0.01).

### 3.5. Transcriptome Profiling of Gene Expression

**Figure 6:**
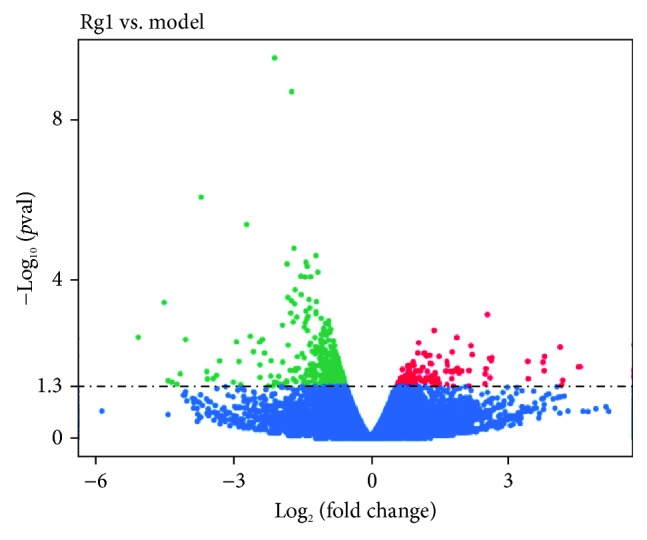
Volcano plot of DEGs in the comparison of Rg1 vs. the model. Red dots indicate differentially expressed genes which are upregulated, green dots indicate differentially expressed genes which are downregulated, and blue dots represent genes with no significant difference.

**Figure 7:**
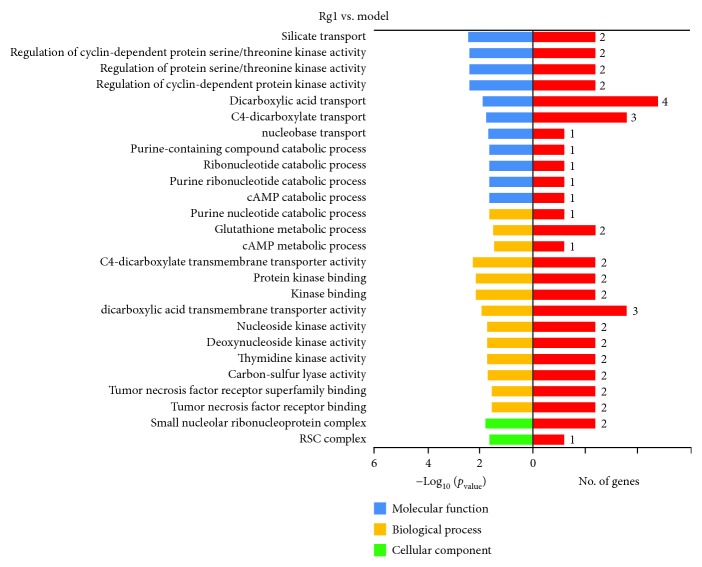
GO functional enrichment analysis. The DEGs between the Rg1 and model groups were classified based on Gene Ontology.

**Figure 8:**
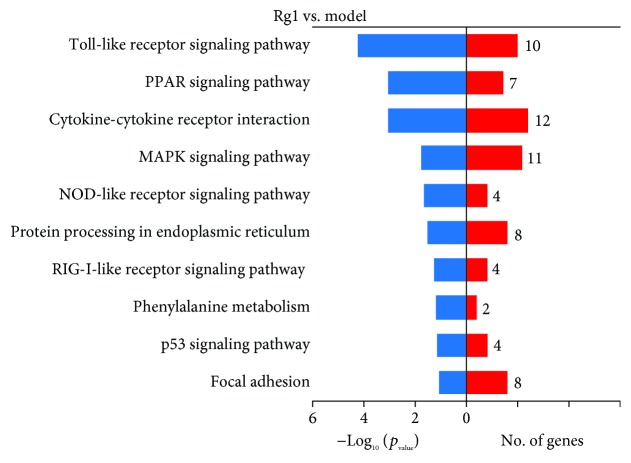
KEGG pathway analysis. The enriched pathways among the DEGs were identified by KEGG analysis.

The results showed that the average of the clean read rate was 97.20%, and the average of the mapping rate was 87.24%. The detailed information of each sample is shown in Supplementary Materials: Table S1. The DEGs were represented in Figure 6. A total of 428 genes were identified as DEGs, of which 105 genes were upregulated, while 323 genes were downregulated. The information of DEGs including the gene symbol and gene description was listed in Supplementary Materials: Table S2. According to the GO classifications, “silicate transport,” “regulation of cyclin-dependent protein,” and “regulation of serine/threonine kinase activity” were the predominant terms in the molecular function category. Meanwhile, “purine nucleotide catabolic process,” “glutathione metabolic process,” and “cAMP catabolic process” were the predominant terms in the biological process category. In addition, we also found “tumor necrosis factor receptor binding” and “tumor necrosis factor receptor superfamily binding” in the biological process category (Figure 7). As shown in Figure 8, we identified ten DEGs in the Toll-like receptor signaling pathway, nine of which were downregulated, whereas one was upregulated. In addition, seven DEGs in the PPAR signaling pathway and twelve DEGs in the cytokine-cytokine receptor interaction were downregulated. We also identified eleven DEGs and four DEGs in the mitogen-activated protein kinase (MAPK) signaling pathway and p53 signaling pathway.

### 3.6. Confirmation of Selected DEG Candidates by RT-qPCR

**Figure 9:**
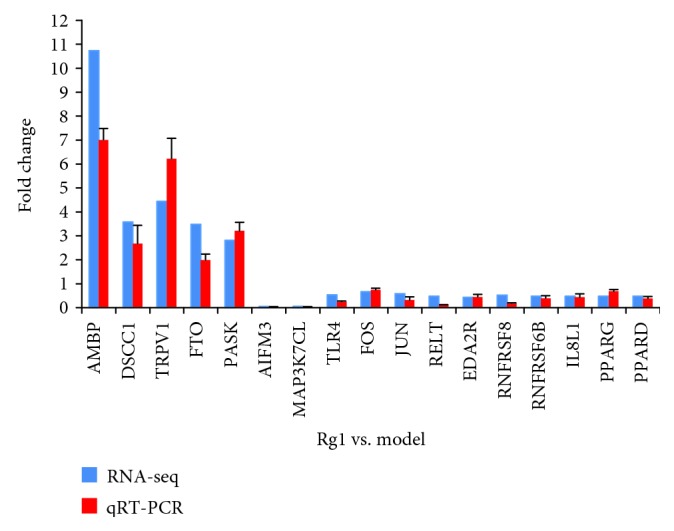
Gene expression determined by RNA-seq and RT-qPCR (*n* = 3). RT-qPCR validation of seventeen differentially expressed genes in the comparison of Rg1 vs. the model. The data were normalized to the expression of *β*-actin, and the fold changes were calculated as Rg1/model.

To validate the RNA-seq results, seventeen DEGs on the transcript level were measured by RT-qPCR. As shown in Figure 9, the tendency of the RT-qPCR results was in accord with the transcriptome sequencing data.

## 4. Discussion

Lymphocytes play important roles in cellular and humoral immune responses in chickens [14]. Because lymphocytes possess many unsaturated fatty acids in their plasma membranes, they are particularly vulnerable to ROS [42]. In the poultry industry, many environmental factors cause overproduction of ROS which may induce oxidative stress, damage the structure of lymphocytes, and suppress the immunity [43]. H_2_O_2_ has been usually experimentally used to stimulate production of ROS in vitro. Similar to the process found in vivo, exogenous H_2_O_2_ traverses the cell membrane; destroys nucleic acid, proteins, and lipid function; wrecks [Ca^2+^]i homeostasis; and activates mitochondria signals, ultimately leading to cell apoptosis [44–47]. In the present study, exposure of lymphocytes to H_2_O_2_ for 4 h significantly caused intracellular oxidative stress. During stress, lymphocytes were seriously damaged since the cell viability was significantly decreased. Oxidative stress also causes increased apoptotic lymphocytes as implied by increased Annexin V-positive and PI-negative cells under stress. The mitochondrial depolarization is considered to be an early stage in an activated apoptotic pathway of mitochondria and often reflected by the increased ratio of JC-1 polymer/monomer fluorescence in cells [28]. In the present study, H_2_O_2_ remarkably decreased mitochondrial membrane potential of lymphocytes, suggesting that mitochondrial apoptosis was activated. [Ca^2+^]i overload induces apoptosis by releasing proapoptotic factors and breaking the mitochondrial respiratory chain [48, 49]. [Ca^2+^]i concentration is often estimated by intracellular Fluo-3 fluorescent intensity [29]. In this study, H_2_O_2_ significantly increased [Ca^2+^]i concentration, which further confirmed that cell apoptosis was increased. Due to the damage of lymphocytes under oxidative stress, suppressed immune responses to vaccination in association with oxidative stress were observed in chickens in our previous study [19].

The present study demonstrated that Rg1 has protective effect on H_2_O_2_-induced damage of chicken lymphocytes as evidenced by increased cell viability and reversed redox status. Antioxidant effect of plant extracts in chicken lymphocytes has been reported in other studies. Zhang et al. observed that *Sargassum* polysaccharide inhibited oxidative stress induced by infectious bursa disease virus in bursal lymphocytes of chickens [50]. Lv et al. found that a polysaccharide from *Agaricus blazei Murill* had antioxidant effect in chicken peripheral blood lymphocytes treated with cadmium [51]. In this study, Rg1 significantly reduced oxidative stress-induced apoptosis and damage of chicken lymphocytes. Rg1-reduced apoptosis of lymphocytes in chickens may be related to recovered mitochondrial membrane potential, as found in mammals [52, 53]. The in vitro findings in this study may explain the protective effect of Rg1 on the immune response against oxidative stress in chickens in previously found in vivo studies [9].

With the chicken genome project completed, genome-wide gene expression analysis has been used in poultry research [54, 55]. In the present study, we used RNA-seq technology to detect gene expressions across the entire chicken genome to provide predictable pathways in the comparison of Rg1 vs. the model. Since Rg1 at 90 *μ*g/mL provides optimal protective effect on cells, treatment of lymphocytes with Rg1 at this dose was used to identify genes that were differentially expressed between Rg1-treated and the model. About 25.5% of DEGs were upregulated and 75.5% of DEGs were downregulated. Interestingly, these DEGs were involved in GO terms related to apoptosis such as regulation of serine-threonine protein kinases, tumor necrosis factor receptor binding, and tumor necrosis factor receptor superfamily binding. In line with the GO enrichment analysis, KEGG pathway analysis revealed that DEGs were involved in the Toll-like receptor signaling pathway, PPAR signaling pathway, MAPK signaling pathway, and p53 signaling pathway, which were associated with regulation of apoptosis.

Tumor necrosis factor alpha (TNF-*α*) has a far wider range than the original described antitumor activity and is one of the most important cytokines in mediating inflammatory and immune responses [56, 57]. The production of TNF-*α* and subsequent binding by TNF receptors trigger a cascade of intracellular processes with diverse effects such as apoptosis in mammals and birds [58, 59]. In the present study, RELT, TNFRSF8, TNFRSF6B, and EDA2R, which are representatives involved in cytokine-cytokine receptor interaction, were downregulated by Rg1. As a member of the TNF receptor superfamily, RELT is able to bind tumor necrosis factor receptor-associated factor 1 and induce cell apoptosis [60–62]. TNFRSF6, which is a well-known member in TNFRSF, combines with Fas ligand TNFSF6 to induce apoptotic cell death in cells that express this receptor molecule [56]. The downregulated genes related to cytokine-cytokine receptor interaction indicated their potential role in reducing apoptotic lymphocytes by Rg1 and were worth of further investigation. Su et al. have observed that Rg1 decreased TNF-*α* production in activated mice macrophage [24]. Consistently, we showed that Rg1 inhibited gene expression of TNF receptors in oxidative-stressed chicken lymphocytes. Other plant extracts such as *Agaricus blazei Murill* polysaccharides were reported to protect against oxidative stress and reduce expression of TNF-*α* in the spleen of chickens, which was similar to our results [63]. Because the genes of the TNF receptor family were decreased in the Rg1 group, it seems that Rg1 protected lymphocytes of oxidative stress by inhibiting production of cytokines related to cell apoptosis.

Toll-like receptors (TLRs) are membrane-bound receptors and play crucial roles in innate immunity by recognizing pathogen-associated molecular patterns and inducing downstream signaling pathways that activate innate immune responses and produce inflammatory cytokines [64–66]. However, TLRs also play an important role in TNF-*α*-induced apoptosis [67, 68]. In this study, nine genes involved in the Toll-like receptor signaling pathway were significantly decreased by Rg1, such as TLR4, FOS, JUN, and MAP2K3. TLR4 is one of the important members of TLRs, and it also recognizes plant-derived molecules such as taxol and ginsenosides [69, 70]. It was demonstrated that ginsenoside Rg1 could enhance immune responses via the TLR4 signaling pathway [71]. However, another study reported that Rg1 could decrease the inflammation factors by inhibiting TLR3 and TLR4 signaling pathways [72]. Other plant extracts such as Astragalus polysaccharide also displayed regulating effect on TLR4 expression in chickens [73]. In view of these results, it is likely that Rg1 suppressed expression of apoptosis-related genes through suppression of TLR4. As inducible transcription factors, the protooncogenes c-FOS and c-JUN can be translated to FOS and JUN, which can compose a heterodimeric complex that interacts with the activator protein-1 (AP-1) binding site and function cooperatively in signal transduction processes [74]. Interestingly, accumulated evidences have implicated that AP-1 transcription factor complexes can positively or negatively modulate distinct apoptotic pathways, depending on the different microenvironments and cell types [75, 76]. Considering that the expression of FOS and JUN was markedly decreased in the Rg1 group, we speculated that downregulated expression of apoptosis-related genes in oxidative-stressed lymphocytes by Rg1 was associated with decreased AP-1.

In addition to the cytokine-cytokine receptor interaction and the Toll-like receptor signaling pathway, we also identified seven downregulated DEGs involved in the PPAR signaling pathway. Nevertheless, ten DEGs were decreased, and one DEG was increased involved in the MAPK signaling pathway by Rg1. A previous study showed that PPAR*γ*, which is a member of PPARs, played an important role in apoptosis of the chicken pancreas [77]. MAPK is a family of serine-threonine protein kinases that is activated in response to various extracellular stimuli and plays key roles in the biological process such as cell apoptosis and cytokine production in chickens [58, 78]. We also identified four DEGs involved in the p53 signaling pathway in the comparison of Rg1 vs. the model. Considering the important role of PPARs, MAPK, and p53 during apoptosis in birds, we speculated that Rg1 might reduce apoptosis of chicken lymphocytes via multiple mechanisms [79].

The present study demonstrated that Rg1 significantly inhibited production of ROS and MDA, decreased apoptosis, and enhanced viability in lymphocytes. The antioxidant property of Rg1 may explain its immune-potentiating effect on birds with oxidative stress as found in our previous study [9]. Numerous DEGs between Rg1-treated and model lymphocytes were identified. Of them, 323 genes were downregulated and 105 genes were upregulated in Rg1-treated cells. The DEGs were involved in Toll-like receptors, PPAR signaling pathway, and cytokine-cytokine receptor interaction. The present study indicated that Rg1 may act as an antioxidative agent to protect cell damage caused by oxidative stress via regulation of gene expression.

## Figures and Tables

**Table 1:** Experimental design.

Groups	*n*	H_2_O_2_ (*μ*mol/L)	Rg1 (*μ*g/mL)
Control	6	0	0
Mode	6	100	0
Rg1-50	6	100	50
Rg1-70	6	100	70
Rg1-90	6	100	90

## Data Availability

The data used to support the findings of this study are available from the corresponding author upon request.

## Supplementary Materials

Supplementary Materials Table S1: summary of the sequencing and the read mapping of the model and Rg1 groups. Table S2: list of the up-/downregulated DEGs in comparison of Rg1 vs. the model. Table S3: sequences of primers for RT-qPCR.
